# Using flawed, uncertain, proximate and sparse (FUPS) data in the context of complexity: learning from the case of child mental health

**DOI:** 10.1186/s12916-018-1079-6

**Published:** 2018-06-13

**Authors:** Miranda Wolpert, Harry Rutter

**Affiliations:** 1 Child Outcomes Research Consortium, UCL and the Anna Freud National Centre for Children and Families, 47 Brunswick Place, London, N1 6EB UK; 2 Centre for Global Chronic Conditions, London School of Hygiene and Tropical Medicine, 15–17 Tavistock Place, London, WC1H 9SH UK

**Keywords:** Child mental health services, outcomes, service development

## Abstract

The use of routinely collected data that are flawed and limited to inform service development in healthcare systems needs to be considered, both theoretically and practically, given the reality in many areas of healthcare that only poor-quality data are available for use in complex adaptive systems. Data may be compromised in a range of ways. They may be flawed, due to missing or erroneously recorded entries; uncertain, due to differences in how data items are rated or conceptualised; proximate, in that data items are a proxy for key issues of concern; and sparse, in that a low volume of cases within key subgroups may limit the possibility of statistical inference. The term ‘FUPS’ is proposed to describe these flawed, uncertain, proximate and sparse datasets. Many of the systems that seek to use FUPS data may be characterised as dynamic and complex, involving a wide range of agents whose actions impact on each other in reverberating ways, leading to feedback and adaptation. The literature on the use of routinely collected data in healthcare is often implicitly premised on the availability of high-quality data to be used in complicated but not necessarily complex systems. This paper presents an example of the use of a FUPS dataset in the complex system of child mental healthcare. The dataset comprised routinely collected data from services that were part of a national service transformation initiative in child mental health from 2011 to 2015. The paper explores the use of this FUPS dataset to support meaningful dialogue between key stakeholders, including service providers, funders and users, in relation to outcomes of services. There is a particular focus on the potential for service improvement and learning. The issues raised and principles for practice suggested have relevance for other health communities that similarly face the dilemma of how to address the gap between the ideal of comprehensive clear data used in complicated, but not complex, contexts, and the reality of FUPS data in the context of complexity.

## Background

There is growing interest in the possibilities posed by the analysis of routinely collected administrative data for the improvement of healthcare, and in particular in the assessment of the impact of services (e.g. [1, 2]). Whilst there has been debate about the challenges of finding the best metrics to use or which analyses are the most appropriate (e.g. [3, 4]), the literature is generally premised on an assumption of the requirement to use high-quality, routinely collected data. However, in many areas of healthcare, the reality is that routinely collected datasets are often of low quality. Data may be flawed, due to missing or erroneously recorded data; uncertain, due to differences in how data items are rated or conceptualised; proximate, in that they are a proxy for the focus of interest; and sparse, in that there may be a particularly low volume of cases for key subgroups. Given the slow pace of advance in routine data capture across many parts of the health system in England (e.g. [5]), it is perhaps best to assume that these datasets may remain flawed, uncertain, proximate and sparse long enough to warrant coining the acronym ‘FUPS’ [6].

Much sophisticated attention and thought has been given to the merits and demerits of different metrics to judge the impact of using routine data in healthcare, alongside calls to support the uptake of new evidence [7–11]. However, there has been less attention given to the properties of the systems in which such data will be used. The prevailing assumption appears to be that such data will be used in systems that may be highly complicated but not necessarily complex. The distinction in this context is that complex systems have key attributes missing from other systems, however complicated, and include emergence (the system has properties greater than the sum of parts not directly predicted from the elements within), feedback (changes reinforce or offset further changes), and adaptation (agents adjust and adapt in response to other agents). Such complex systems have been characterised as “*a collection of individual agents with freedom to act in ways that are not always totally predictable, and whose actions are interconnected so that one agent’s actions changes the context for other agents*” [12]. There are increasing calls for a greater consideration of the implications of the complexity of the healthcare system in relation to both research and practice [12, 13].

This paper considers the use of FUPS data in the context of the complex system that is child mental health. Part case study and part vision for the future, we draw on learning from the use of a national dataset of child mental health outcomes following contact with specialist mental health services. The learning is relevant for any healthcare system that faces the gap between the ideal of comprehensive, clear data used in complicated but not complex contexts, and the reality of FUPS data used in contexts of complexity. In particular, we consider both how FUPS data can and should be used to help evaluate aspects of a complex system as well as to help influence behaviours in such a system.

In terms of using FUPS data to evaluate outcomes in complex systems, the paper explores how analysis of FUPS data can in part be treated like a historical investigation where partial remnants and sources are used to consider the complex reality they relate to, but cannot fully capture it, in order to build up narrative arguments and hypotheses that can be explicitly contested and debated within a system.

With regard to using FUPS data to help influence behaviours in complex systems, the paper examines the potential to draw on learning from a range of disciplines to consider some key factors that might influence the use of findings from data, whether FUPS or not; for example, findings from cognitive psychology that suggest people are most likely to reject data that challenge existing assumptions [14], and findings from sociology that suggest power elites may look to protect their vested interests [15]. The implications of these findings for the use of FUPS data are explored and the differentiated standards of proof derived from legal frameworks to aid appropriate use are considered.

The philosopher and urban planner Donald Schon famously drew the distinction between the “*high ground,* [where] *manageable problems lend themselves to solution through the use of research-based theory and technique*” and the “*swampy lowlands,* [where] *problems are messy and confusing and incapable of technical solution…*” [16]. This paper is premised on the belief that decision-making in complex healthcare systems occurs in the ‘swampy lowlands’ of practice, where decisions are necessarily made every day, whether or not there are good quality data to support them, and in the context of a complex network of existing beliefs, relationships and assumptions; it is in this context that we consider the use of FUPS.

## Child mental healthcare: an example of a complex system

### Emergent properties: structural

A diverse range of providers, such as health providers (both in primary, secondary and tertiary care), voluntary sector providers, social care and, increasingly, schools, work to support child mental health across various agencies [17]. A lack of quality data to support this system is perceived as a major issue, with services having been described as “*working in a fog*” [18]. The central flow of child-level data to NHS Digital was only initiated in mid-2016, following a decade-long process of implementing a national dataset for child mental health and, to date, returns are still limited in quality and quantity. Whilst there has been a policy and practice commitment with regards to the need for integrated cross-agency collaboration and co-ordination, and appropriate use of data for more than a decade, fragmentation and confusion have been the described emergent defining features of the system [18, 19].

### Agents within the system

Agents within the system include (but are not limited to) children and young people with mental health issues, their parents and family members, school staff, primary care providers (including GPs and school nurses), specialist mental health providers (both voluntary, statutory and independent), care providers, trainers of specialist mental health providers, professional bodies of specialist mental health providers, pharmaceutical companies, psychological treatment developers, policymakers, politicians, civil servants, data analysts, commissioners, researchers, service support and improvement organisations (e.g. NHS Improvement), service review and assessment organisations (e.g. Care Quality Commission), and guideline developers (e.g. National Institute for Health and Care Excellence (NICE)).

### Emergent properties: cultural

Whilst there are debates between agents within the system about the causes and nature of mental health problems and the best approaches to take, a dominant and consensual narrative is the need for earlier intervention and, in particular, faster and easier access to specialist services (e.g. [17, 20]). There is also a shared emphasis on the need to address the stigma surrounding mental health issues that are seen as blocking people from accessing help (e.g. the ‘Time to Talk’ campaign). Public discourse, guidance to policymakers and the wider public tend to emphasise the fact that many mental health problems in adulthood are reported to have originated in childhood; 75% of adult mental health problems start before the age of 18 [21], one in four children has a mental health problem at any one time [22], and these mental health problems may have long-term negative impacts if not successfully addressed [23].

The existence of a range of evidence-based treatments, whose effectiveness is based on comparison of differences in group means in randomised control trials, are emphasised. Thus, websites offering information to the public stress the advantages of accessing specialist help and suggest that, without help, children and adolescents will fail to improve. For example, “*Like other medical conditions, anxiety disorders tend to be chronic unless properly treated. Most kids find that they need professional guidance to successfully manage and overcome their anxiety*” [24]. These data and statements are regularly mobilised as part of an argument for more services and to encourage children and their parents to seek help sooner rather than later.

Less quoted in both public and professional discourse is what is known about rates of spontaneous improvement (which might in fact be better defined as improvement that occurs without professional input, since it may include interventions and input from many other agents in the system). The rates of non-professionally mediated improvement for key difficulties may be as high as 60% for adolescent depression [25]. Whilst a number of systematic reviews have identified effective prevention interventions for mental ill health in children, with moderate effect sizes across diverse populations [26–28], there is still a need for further evidence of the best ways forward in terms of early intervention or prevention and how to ensure there are no unintended harms [29, 30]. There is also little discussion of likely rates of recovery or non-recovery following treatment [31]. Further, no NICE guideline for child mental health contains any reference to non-response or how children should be supported if they do not respond to treatment.

### Current system challenges: implications for feedback and adaptation

The complex and dynamic child mental health system is particularly beset at the moment with a range of challenges. The system faces a heady mix of increased prevalence rates [32, 33], cuts across a number of the diverse range of services [34], and increased pressure on both schools and specialist services [35, 36]. There has also been more public scrutiny and concern; in the last 5 years, there have been over five national reviews, three health committee reports, several policy documents and a Green Paper currently out for consultation. The outputs of these reports tend to repeat the same messages of the need for better coordination, earlier intervention and more resources. Given this context, it is anticipated that many stakeholders will be particularly sensitive and wary of any data that might undermine existing discourses on the benefits of increased provision and the need for more resources. In particular, there are likely concerns that any suggestion of poor performance on the part of current services may lead to further cuts and loss for children and families.

### An attempt to ‘transform’ the system

A major initiative within the system was the Children and Young People’s Improving Access to Psychological Therapy (CYP IAPT) programme, led by the Department of Health and NHS England, and involving geographical partnerships between NHS providers, local authorities and voluntary sector providers across five areas (London and the South East, the North West, Oxford/Reading, Yorkshire, Humber and the North East, and the South West). The programme sought to embed best practice in child mental health provision by focussing on specific elements of participating services, namely helping them work effectively in partnership with children and young people so that they were active in shaping their local services; supporting services to develop a culture of reflective practice and accountability; improving the workforce through training in best evidence-based practice; developing mechanisms to deliver frequent outcome monitoring to help the therapist and service user work together in their session, and to help supervisors support therapists in improving outcomes; and supporting local areas in improving the infrastructure they use to collect and analyse data to assess whether children and young people are getting better.

The premise of CYP IAPT was to train a selection of practitioners, supervisors and managers, alongside providing additional resources for infrastructure and building regional and national collaborations to support best practice. In this way, the aim was to maximise limited resources and facilitate the embedding of sustainability. Specific training programmes were developed for both practice and supervision in cognitive behavioural therapy for anxiety, parent training for behavioural difficulties in children under the age of 9, systemic family therapy for eating disorders, conduct disorders and depression, interpersonal psychotherapy for adolescent depression, leadership, service development, supervision skills and service transformation skills, and enhanced evidence-based practice.

The programme was rolled out over 4 years (2011–2015) and sought to embed seven key principles in child mental health services, namely to support whole service transformation through leadership; to improve access through self-referral; to work in partnership with the young person and their parent/carer in service delivery and design; to deliver evidence-based psychological treatments; to deliver outcomes-focused psychological treatments; to work in partnership with the young person and their parent/carer throughout treatment; and to provide supervision to support the delivery of evidence-based, service user-informed and outcomes-informed practice. The programme involved directly training over 1000 clinicians and service managers in evidence-based approaches and leadership [6]. The vision was that these trained staff would lead service transformation and more effective practice within their organisations.

A key aspect of this initiative was the emphasis on the collection of child- and parent-reported questionnaire data throughout the course of treatment that sought to capture change in symptoms, wellbeing, functioning or achievement of goals during the course of treatment [6]. Between 2011 and 2015, the lead author (MW) and colleagues were commissioned first by the Department of Health and then NHS England to agree which data to collect and then to collect and analyse routinely collected CYP IAPT data with a particular focus on the child- and parent- reported outcome data [6].

An outcomes and evaluation group was convened, chaired by MW. This group oversaw measure selection and the approach to data collection; chose measures based on review of psychometric properties, feasibility, utility, compatibility and cost; advised on how to implement routine outcome measures and how to report findings; and consulted with wider networks and held regular public consultations on measures to include in the dataset [6]. Out of this process, 21 child-report scales and 15 parent-report scales were used to cover the range of problems seen in child mental health services. Since there was no national data flow, patient-level data from participating sites were submitted quarterly using an agreed data specification. Data were uploaded via secure data handling to a data storage provider and collated centrally. In the first year of a site’s involvement in the initiative, data were largely sent from those involved directly in the training; from the second year of involvement onwards, data were sent from all practitioners across the partnership. Data collected included demographic information and outcome and experience measures, with a particular emphasis on child and parent reports [6].

## CYP IAPT data: an example of a FUPS dataset

**Fig. 1:**
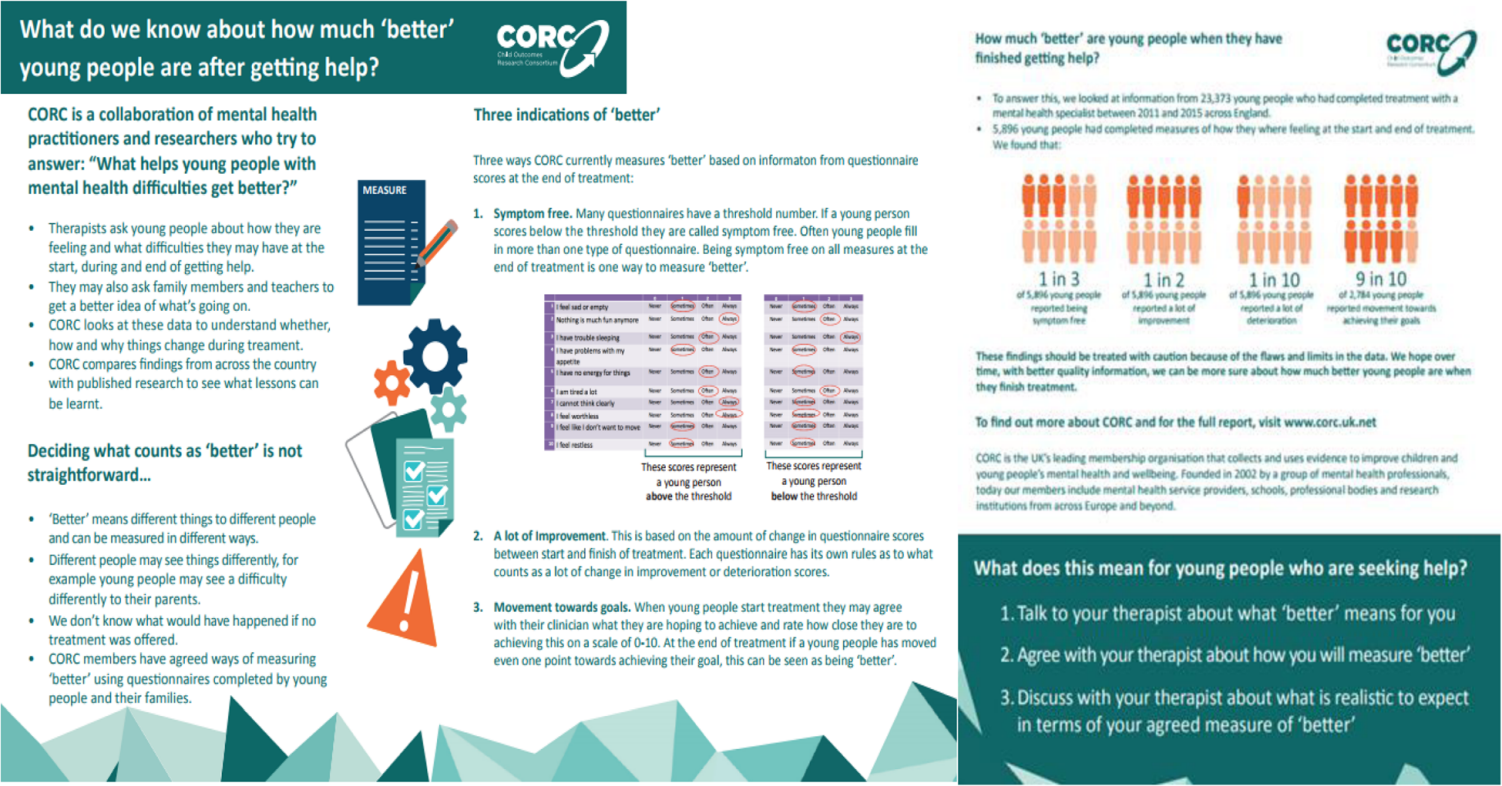
Diagram to show data captured (and lost) in the project

**Fig. 2:**
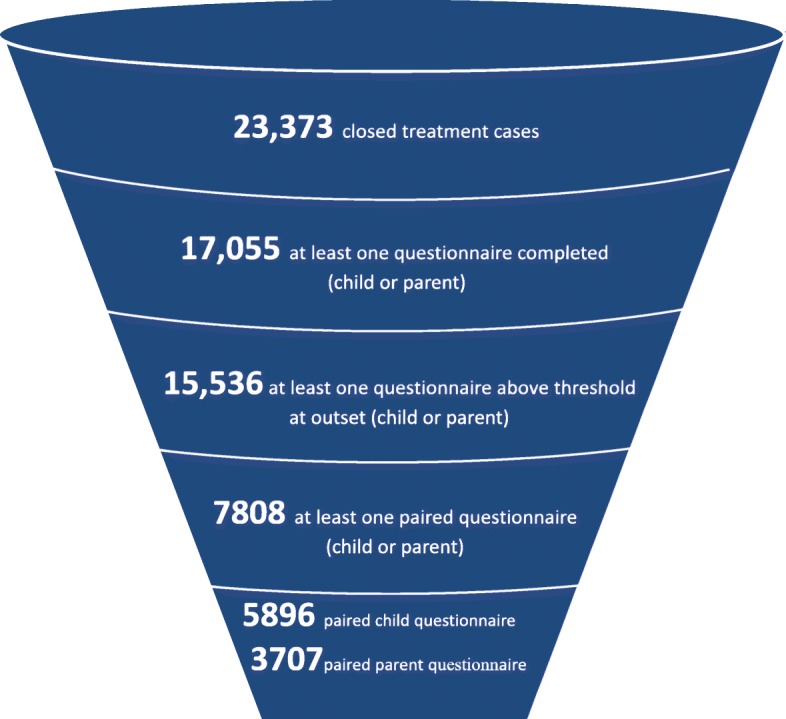
Infographic explaining findings to children and families

The CYP IAPT data can be seen as an example of a FUPS dataset. Out of the approximately 23,000 cases of completed treatment^1^ in this period (April 2011 – June 2015), approximately 8000 had paired child and/or parent-reported data relevant to outcomes (~ 6000 had child-report data and ~ 4000 parent-report data) (Fig. 1). Based on these data, 52% of child-report data and 40% of parent-report data showed ‘reliable improvement’,^2^ 9% of child-report data and 9% of parent-report data showed ‘reliable deterioration’,^3^ and 36% of child-report data and 26% of parent-report data showed ‘recovery’^4^. These findings were summarised in an infographic (Fig. 2), which was designed to share this information with children and families (see discussion on the use of FUPS data below).

### CYP IAPT data fit the criteria for FUPS

#### Flawed

There is a high degree of missing data. The sample of approximately 8000 cases with outcome data available for analysis represents just under half of the approximately 16,000 cases who had completed treatment. This represents less than a third of the over 23,000 people who completed treatment during this period. The missing data are unlikely to be missing entirely at random, although it is hard to know how the different skews might operate at different points. Taking the paired data subsample, for example, it could be that those who feel most positive about treatment are more likely to complete a second questionnaire, leading to a potential inflation of positive outcomes, or it may be that those who quickly improve stop coming and thus are less likely to complete a second questionnaire, leading to a potential underestimation of positive outcomes [6].

#### Uncertain

A total of 14 questionnaires were used (10 child completed, 4 parent completed) covering anxiety, depression, trauma, behavioural problems, attention problems, general functioning and general distress [37]. Different questionnaires use different approaches to calculate ‘clinical’ thresholds, all involve a high degree of measurement error and there is known to be a low correlation between child, parent and teacher perspectives even when completing the same questionnaire [38–41].

#### Proximate

Without a biological marker, the field is largely reliant on changes in questionnaire scores over time. Even if change in scores is agreed as a suitable proxy of change in mental health status the data are still proximate to the issue of ultimate issue interest, i.e. the impact of service provision. Child mental health problems follow a fluctuating course. To determine if someone would have improved or worsened without the treatment being offered it is necessary to have a counterfactual, which is not available in these data.

#### Sparse

For key subgroups, data are particularly sparse. For example, the vast majority of children in this sample were from white British ethnic communities, with only 18% of the full sample being from black, Asian and minority ethnic (BAME) groups. Whilst this may be an appropriate reflection of the populations covered, this means that, for the sample with paired outcomes, the numbers from BAME groups are too small to be able to undertake viable subgroup analyses, which may be important in the light of findings that BAME groups may differ in access and use of services [42].

## Analysing FUPS data: the example of CYP IAPT

Given the FUPS nature of the data and the complexity of the system, careful thought was given to how the data can be analysed and used. The responsibility to steer between over- and under-interpretation of any findings was felt very keenly. It was recognised that these data were not being considered in a neutral space. The current discourse and challenges within the child mental health system meant that consideration of these data might have important implications for future service development and it was clear that the data could not provide a comprehensive insight into the complex reality of the full system. In examining and using data such as these, it was felt relevant to draw on learning from other disciplines that need to use FUPS data.

### FUPS data for evaluation: considering data as historical fragments

Historians draw on FUPS data all the time. As an historical record, Pepys’ diary [43] is flawed in that it is not representative of all people living in this period, uncertain in that the author is a highly unreliable narrator, proximate in that, even where Pepys is most honest, it is still refracted through his perception, and sparse on some of the key details we might like to know about. This does not stop it being a source much pored over for insights into seventeenth-century life. The scientific paradigm has developed, at least partly, to try to manage complexity in such a way as to make findings more generalisable, and this paper is not an attempt to undermine that paradigm. Rather, we are suggesting we can draw on FUPS data to generate and consider hypotheses and make arguments that can helpfully contribute to the discourse in a complex system.

This is in contradistinction to the current approach to FUPS data, which is generally attacked from two standpoints within the child mental health field. First, many agents in the system, such as clinical researchers, are influenced by the dominance of using biomedical evidence to inform healthcare and have been trained to interrogate data using the highest standards of traditional scientific evidence. Second, and in distinction, other agents, such as many talking therapists, have been trained to contest data using a different but equally demanding set of criteria, pointing to limitations in being able to capture the complexities of human experience. In both instances, however, as noted above, criticisms of the flaws of the data and a tendency to dismiss them may be particularly evident where such data challenge strongly held convictions or interests.

### Transparency and triangulation

In order to treat the data more as a historical source than as a sacred source of truth, three key principles of analysis are suggested and were used in this dataset. (1) Treat data as a fragment of the whole and be honest and upfront about its limitations – it is crucial to present data in such a way as to convey any limitations to the validity, reliability and generalisability of data, stemming from its FUPS characteristics. (2) Be transparent in all analyses and avoid ‘black box’ statistics – it is important to use precise and neutral language and to keep the sophistication of analysis commensurate with the flaws in the data. Thus, it is recommended to use very simple and transparent statistical approaches to allow maximum opportunity for debate and consideration. (3) Triangulate the findings with other information. It is important to remember that these data are being considered within the context of the ‘swampy lowlands’ of practice. In trying to make sense and reflect on information available, it is key to consider it in the context of other information to see what supports or undermines the findings from these particular FUPS data.

These principles, and how they were employed in relation to the CYP IAPT dataset, are outlined in Table 1 below.

**Table 1:** Outline of key proposed principles for analysing flawed, uncertain, proximate or sparse (FUPS) data and how they were employed in CYP IAPT

	Principles for analysing FUPS data	How instantiated in relation to CYP IAPT FUPS data
1	Treat data as a partial remnant Present data in such a way as to convey any limitations to interpretation stemming from its FUPS characteristics	• Introduced notion of FUPS data at start of report • Kept reiterating limitations of data at relevant points in report • Presented flow diagram of data loss • Chapter on implications of missing data and hypotheses as to potential impact • Undertook and invited blogs and responses on potential interpretations and limitations
2	Transparency of analyses: avoid ‘black box’ statistics Ensure that all use of data follows principles of transparency and clarity	• Included detail of questionnaires • Ensure all axis labels on graphs are factual (what was questionnaire) rather than interpretive (performance or quality of care) • Did not use terms ‘significance’ or ‘performance data’ • Detailed descriptions given on all metrics • Clarified where different measures had different thresholds or other key metrics • Kept different groups separate, i.e. parent and child reports considered separately • Included raw numbers in the report and reiterated denominators regularly. All statistical techniques used in the report were clearly explained and not complicated • No ‘black box’ techniques used • Very clear that data not collected as part of a trial so could not be taken as an evaluation of the programme itself
3	Triangulation Consider the data in the context of other information to see what supports or undermines the findings from these particular FUPS data	• Reviewed other relevant information from the literature • Contextualised against information from other areas of healthcare • Made clear that status quo may not be safer or more effective than alternative

## Using FUPS data: the example of CYP IAPT

When we shared findings with a group of respected and experienced child health academics, they suggested that these analyses should not be shared at all as the data were too flawed and any analysis might lead to misleading conclusions. After much careful reflection and debate, the decision was made to go ahead with presenting the analysis, but to do so in the context of stressing the FUPS qualities of the data and considering how best to share it in the light of the specific challenges and issues facing the complex dynamic system of child mental health care and with due attention to likely feedback and adaptation processes. We looked to another discipline, that of the law, for guidance on the use of FUPS data for decision-making.

### Standards of evidence – the legal perspective

The courts deal with FUPS data all the time. Many jurisdictions apply different standards of evidence depending on a range of factors, one of which may be the consequence that hangs on any decision. UK law, for example, lays out three different standards of evidence that will be needed for different decisions, ranging from the highest standard of proof, which is ‘beyond reasonable doubt’ (used, for example, in criminal cases), to the next standard, which is ‘on the balance of probabilities’ (used in civil cases), and finally to concepts used where a decision needs to be made between competing accounts but the risks and benefits are such that the court will base its decision on lower standards of proof such as ‘a reasonable chance’, ‘substantial grounds for thinking’ and ‘a serious possibility’ as means of describing the likelihood [44]. These are frequently used in cases involving resolution of competing claims over contracts, for example.

In medical academic literature considering evidence is sometimes discussed as if there is only one standard of evidence, which set up scientific experiments with the search for a definitive answer (closest to ‘beyond reasonable doubt’). This may be appropriate for some decisions, such as the introduction of a new drug. However, it may be less appropriate for making a decision in the swampy lowlands, such as between funding options when a decision has to be made one way or the other, and there is therefore a need to use the best available evidence, even if that evidence is of poor quality [45, 46].

### ‘Reasonable chance’ as a basis for change

Healthcare is naturally a very conservative profession and the evidence alluded to earlier suggests the system will naturally adapt to continue to practise along well-worn and traditional grooves, regardless of new emerging evidence, even when of high quality [7].

Historical data and findings from cognitive psychology suggest that agents in the system are more likely to apply very high standards of evidence to new initiatives and those that challenge their beliefs and status, than to old or traditional practice, regardless of the fact that the latter may be based on historical precedence alone [7]. This is likely to result in healthcare professionals overestimating the risks of trying something new (including stopping doing something that has been found to be ineffective) and underestimating the risks of continuing to do what they have always done. A range of initiatives has been developed to try to address this tendency (for example, [47]).

With regard to using FUPS data, agents in the system should be encouraged to apply standards of evidence appropriate to the decision needing to be made. This may sometimes require only the standard of ‘a reasonable chance’ where a view of the risks and opportunities of action and inaction are carefully considered. Helping agents examine the risks and opportunities in an even-handed way, which takes into account the likely existing biases in approach to such data, is a key element of the appropriate use of such data to inform potential system change.

### Opening up conversations

In order to try to open up conversations on the findings, and rather than inappropriately treating them as definitive, it was agreed not to issue a press release or seek to feed headlines. Instead, we decided to blog on the topic and invite comments from others in the field to elicit debate and to examine if the findings met the criteria for ‘reasonable chance’. A series of regional debates was convened involving a range of stakeholders (including children and young people with experience of service use, commissioners, policymakers and service providers). We invited a panel that included, as a minimum, a young person with experience of service use, a specialist mental health provider, and a commissioner to comment on the report. We also agreed to frame the conversations as safe spaces to be curious about what these findings might mean.

We were acutely aware that the data we were drawing on were ‘FUPS’. However, we were also aware that it is easy to dismiss uncomfortable findings or hypotheses arising from them due to the FUPSness of the data. Thus, we sought to facilitate conversations using a MINDFUL framework [6]. This involved the use of three principles in this context. (1) Encourage curiosity – it is vital to help stakeholders to maintain curiosity. This involves finding ways to help stakeholders to challenge their own and colleagues’ confirmatory biases, and to apply the same standards of scrutiny to analytic findings that support prior beliefs as to analytic findings that are uncomfortable or not wished for. This includes finding ways to help maintain this stance over time by the development of long-term safe space and relationships. (2) Apply the standard of ‘a reasonable chance’ rather than ‘beyond reasonable doubt’, drawing on how it meshes with existing narratives and how it triangulates with other information. (3) Encourage action – it may be important to help relevant stakeholders to consider possible initiatives that, even if not definitively indicated, may do more good than harm and challenge the assumption that change is always riskier than the status quo. Again, the focus needed to be on long-term change. These principles and how they were employed in relation to the CYP IAPT dataset are outlined in Table 2.

**Table 2:** Outline of key principles used for use of flawed, uncertain, proximate or sparse (FUPS) data in the Children and Young People’s Improving Access to Psychological Therapy (CYP IAPT) context

	Principles for facilitating discussion of FUPS data	How instantiated in relation to CYP IAPT FUPS data
1	Curiosity Help stakeholder to challenge their own and colleagues’ confirmatory biases, and to apply the same standards of scrutiny to analytic findings that support prior beliefs as to analytic findings that are uncomfortable or not wished for; encourage stakeholders to maintain curiosity	• Ensured range of perspectives present to encourage debate and crucially included young people themselves, providers and commissioners as three key groups • Set clear ground rules for conversations (e.g. no point scoring, atmosphere of general interest, welcome critical thinking, focus on possible next steps and options that can aid best practice) • Ensured enough time to reflect and absorb the information – allowed time for questions and debate • Whilst introduced notion of FUPs, data did not allow that to be the only thing discussed and encouraged discussion of alternative explanations, e.g. FUPS data leading to negative skew in outcomes considered against possibility it has led to positive skew in outcomes • Invited reflection on other sources of information that either supported or challenged these findings, including from stakeholders’ lived experience as well as from published literature • Facilitated conversations between different stakeholders to consider any differences in perspective • Invited stakeholders to predict what results were prior to seeing results • Encouraged stakeholders to discuss reasons for prediction • Encouraged discussion of reasons for disparity between prediction and findings
2	Apply the standard of ‘clear and convincing evidence’ rather than ‘beyond reasonable doubt’ drawing on how it meshes with existing narratives and how it triangulates with other information	• Encourage consideration of what can be done with the available evidence • Introduced findings from other areas of healthcare for context and consideration • Encouraged consideration of current use of other forms of evidence
3	Encourage action Help relevant stakeholders to consider possible initiatives that, even if not definitively indicated, may do more good than harm and challenge the assumption that change is always riskier than the status quo	• Encouraged discussion of potential initiatives drawing on those findings that could be trialled • Encouraged sharing of current practice development that aligns with potential implications of findings • Supported networks of practitioners and others taking ideas forward and checking in on progress and recognise that change takes time and draws on long-term relationships • Supported teachers to consider if this might support idea that not everyone is better if seen by specialist services, so still may need support in schools • Supported initiatives that focus on and how to address ongoing needs when, at the end of treatment, the child has not achieved reliable improvement or recovery, e.g. establishment of a long-term conditions clinic that allows people to opt in for up to 2 years post treatment

### Impact in child mental health

As is to be expected with a complex dynamic system, it is hard to disentangle the impact of opening up conversations on the FUPS CYP IAPT data findings, and our perspectives (particularly those of MW, who led on some of these conversations) will themselves be partial, unreliable and flawed. With these caveats in mind, we would share the following reflections on emergent properties from the system which clearly are multiply determined and influenced by a range of factors, and where cause and effect reverberate around the system with impact from both feedback and adaptation. First, it is noted that the findings from this FUPS dataset are now being used nationally to help services benchmark their outcomes (in the context of all the caveats above), which has been welcomed as a way for service providers and commissioners to consider and agree realistic standards for local services [48]. A debate has opened up about how to end specialist mental health treatment in the context of a child having ongoing difficulties. Initiatives that have developed include the development of long-term conditions clinics specifically for mental health issues that allow people more flexible re-entry but also allow earlier case closure in the recognition that greater improvement may be unlikely [49]. Some clinicians have started to talk more openly about likely improvement rates of treatment and to use this with their clients [31]. We would contend that this suggests that, if the principles we outline above are applied, there is a possibility that a complex system will consider and respond to even challenging FUPS data, and that this may be true across a variety of healthcare contexts.

## Conclusions

Datasets that can be considered FUPS are likely to exist in many domains of complex and dynamic healthcare systems. There are clearly dangers of over-interpretation of such data, but there may also be dangers of non-use, which allow stakeholders to use the FUPSness of the data to ignore potentially important but uncomfortable findings and hypotheses. This paper has presented some suggested principles for the use of FUPS data, drawing on both historical and legal disciplines to try to move beyond the biomedical model as the only model of evidence. The redoubtable historian EH Carr once noted that, rather than history being “*a hard core of facts leading to a range of interpretations*”, historical debate could be seen as “*a hard core of interpretation surrounded by a pulp of disputable facts*” [50]. Debates in complex healthcare systems take place within existing, highly charged discourses involving hard cores of interpretation formed over many decades. FUPS data are clearly disputable facts, but they can be drawn on as a form of evidence to aid decisions in the swampy lowlands of practice.

## Abbreviations

BAME: Black, Asian and Minority Ethnic
CYP IAPT: Children and Young People’s Improving Access to Psychological Therapy
FUPS: Flawed, Uncertain, Proximate and Sparse
NHS: National Health Service
NICE: National Institute for Health and Care Excellence

## Footnotes

1 Defined as two or more contacts and where at least one was not defined as assessment only for closed cases.

2 Reliable improvement = amount of improvement between first and last collected score is greater than likely due to measurement error on at least one subscale of a questionnaire AND amount of deterioration of first and last collected score is NOT greater than likely due to measurement error on any subscale.

3 Reliable deterioration = amount of deterioration between first and last collected score is greater than likely due to measurement error on at least one subscale regardless of amount of improvement on any other subscale.

4 Recovery = at least one subscale score above threshold at outset and, at closure, all scores below threshold.
